# *Cannabis sativa* and the endocannabinoid system in cervical cancer: molecular targets and mechanistic insights from preclinical models-narrative review

**DOI:** 10.1186/s42238-026-00476-y

**Published:** 2026-08-10

**Authors:** Sanele Mhlungu, Malcom Steinberg, Motshedisi Sebitloane, Mlungisi Ngcobo, Nceba Gqaleni

**Affiliations:** 1 Discipline of Traditional Medicine, University of KwaZulu-Natal, Durban, South Africa; 2 Faculty of Health Sciences, Simon Fraser University, Burnaby, BC Canada; 3 Discipline of Obstetrics and Gynaecology, Nelson R. Mandela School of Medicine, Durban, South Africa; 4 Africa Health Research Institute, K-RITH Tower Building, Durban, South Africa

**Keywords:** *Cannabis sativa*, Endocannabinoid system, Cannabinoids, Cervical cancer, Cannabinoid receptors, Preclinical studies

## Abstract

**Introduction:**

Cervical cancer is among the major causes of cancer-related mortality, particularly in low- and middle-income countries where access to effective screening and treatment is limited. The endocannabinoid system (ECS), which comprises of cannabinoid receptors (CB1 and CB2), non-classical targets (TRPV1, GPR18 and GPR55), endogenous ligands, and metabolic enzymes, has emerged as a potential therapeutic target in several malignancies, including cervical cancer. *Cannabis sativa (C. sativa)*–derived phytocannabinoids, notably cannabidiol (CBD) and Δ⁹-tetrahydrocannabinol (Δ⁹-THC), interact with ECS components and may modulate key cancer-related pathways. This review aims to critically assess in vitro evidence on the effects of *C. sativa* phytochemicals in cervical cancer and to examine the underlying mechanisms associated with endocannabinoid system modulation.

**Methods:**

A narrative literature search was conducted using PubMed, Scopus, Web of Science, ScienceDirect, and Google Scholar. Relevant peer-reviewed English-language studies investigating *Cannabis sativa*, cannabinoids, or ECS components in cervical cancer models were included. Five studies met the inclusion criteria. Only 5 studies were eligible for this study.

**Key findings:**

Available in vitro evidence indicates that C. sativa extracts and phytocannabinoids exhibit cytotoxic and mechanistic effects in cervical cancer cell lines. These effects involve both ECS-dependent and independent pathways, including TRPV1 activation, modulation of MAPK signaling, induction of TIMP-1, reactive oxygen species (ROS) generation, mitochondrial dysfunction, and apoptosis. However, evidence also suggests context-dependent and potentially opposing roles of ECS signaling, including cytoprotective effects mediated by CB1/CB2 receptors.

**Conclusion:**

Current evidence is limited to a small number of in vitro studies and remains insufficient to support conclusions regarding therapeutic efficacy. The observed effects are exploratory and highly context-dependent. Further research, including in vivo validation and studies in HPV-driven models, is required to determine the biological and clinical relevance of ECS modulation in cervical cancer.

## Introduction

Cervical cancer remains a major global health challenge, particularly in sub-Saharan Africa, where limited access to effective screening and treatment contributes to high mortality rates (Singh et al. 2023; Sung et al. 2021). In low and middle-income countries, an estimated two in three women aged 30–49 have never been screened for cervical cancer (Bruni et al. 2022). In South Africa, according to the GLOBOCON 2020 statistics, cervical cancer is the third most common cancer, accounting for 18.7% of new cases annually, and is the second leading cause of death, with a mortality of 10.3%, when compared to other types of cancer. These statistics highlight persistent gaps in prevention, early detection, and effective disease management.

The etiology of cervical cancer is strongly associated with persistent infection by high-risk human papillomavirus (HPV) subtypes, particularly HPV 16, 18, 31, 33, and 45, which are detected in over 90% of cases (Okunade 2020; Ye et al. 2024). Cervical oncogenesis is primarily driven by the viral oncoproteins E6 and E7, which promote malignant transformation through the degradation of tumor suppressor proteins such as p53 and retinoblastoma (Rb). This results in uncontrolled cell proliferation, genomic instability, and evasion of apoptosis (Pal and Kundu 2020; Yugawa and Kiyono 2009). While prophylactic HPV vaccines and screening programs have significantly reduced disease burden in high-income countries (Abbas et al., 2024), their impact remains limited in resource-constrained countries due to challenges related to accessibility, healthcare infrastructure, and public health implementation (Aleezada et al. 2025; Denny et al. 2017).

Current treatment strategies for cervical cancer include surgery, radiotherapy, and chemotherapy, administered either alone or in combination depending on the disease stage (Dicu-Andreescu et al. 2023; Kumar et al. 2018). Early-stage disease is often managed surgically, while locally advanced cases typically require concurrent chemoradiotherapy (Akiseku et al. 2025; Lin et al. 2025; Mulugeta-Gordon et al. 2026). Although these strategies have improved survival outcomes, they are associated with considerable limitations, including systemic toxicity, treatment resistance, recurrence, and reduced quality of life (Davidson 2011). Moreover, access to advanced therapies remains restricted in many low-resource settings, underscoring the urgent need for novel, cost-effective, and targeted therapeutic approaches (Gopalkrishnan and Karim 2025; Srinivasan 2024).

In this context, the endocannabinoid system (ECS) has emerged as a promising area of investigation in cancer biology. The ECS comprises cannabinoid receptors (CB1 and CB2), endogenous ligands, and enzymes responsible for their synthesis and degradation, forming a complex signaling network that regulates key physiological and pathological processes (Lu and Mackie 2016; Stasiulewicz et al. 2020). Accumulating evidence indicates that the ECS plays a role in tumor development and progression by modulating cell proliferation, apoptosis, angiogenesis, metastasis, and immune responses (Liu et al. 2020; Moreno et al. 2019, 2020; Sen et al. 2025; Taylor et al. 2020). Despite its involvement in key cancer-related pathways, the ECS remains relatively underexplored as a therapeutic target in cervical cancer, particularly in the context of HPV-driven malignancies, providing a strong rationale for further investigation. 

*Cannabis sativa* (*C. sativa*) and its bioactive phytochemicals, particularly Δ9-tetrahydrocannabinol (THC) and cannabidiol (CBD), share structural and functional similarities with endogenous cannabinoids, enabling them to interact with the ECS (Schanknecht et al. 2023). These interactions can modulate multiple signaling pathways associated with anticancer activity, including apoptosis induction (Suttithumsatid et al. 2023), inhibition of tumor growth (Yang et al. 2020), suppression of angiogenesis and metastasis (Bachari et al. 2023, 2024). This review aims to critically evaluate the currently available in vitro evidence on the effects of *Cannabis sativa* phytochemicals in cervical cancer and to examine mechanistic insights associated with the endocannabinoid system modulation.

## Literature search strategy

A narrative literature search was conducted on electronic databases including PubMed, Scopus, ScienceDirect, Web of Science, and Google Scholar, covering studies published between 2000 and June 2025. Peer-reviewed, English-language articles were included if they investigated *Cannabis sativa*, its phytochemicals, or components of the endocannabinoid system in the context of cervical cancer and provided experimental evidence from in vitro and or in vivo models. Only full‑text studies with sufficient methodological detail to allow evaluation of study design and outcomes were considered. Non‑English publications, non‑peer‑reviewed literature, conference abstracts without full‑text access, duplicate records, studies of inadequate methodological quality, and studies unrelated to *C. sativa*, cannabinoids, or the endocannabinoid system, including those focused on non-cancer models or cancer types other than cervical cancer, were excluded.

**Fig. 1:**
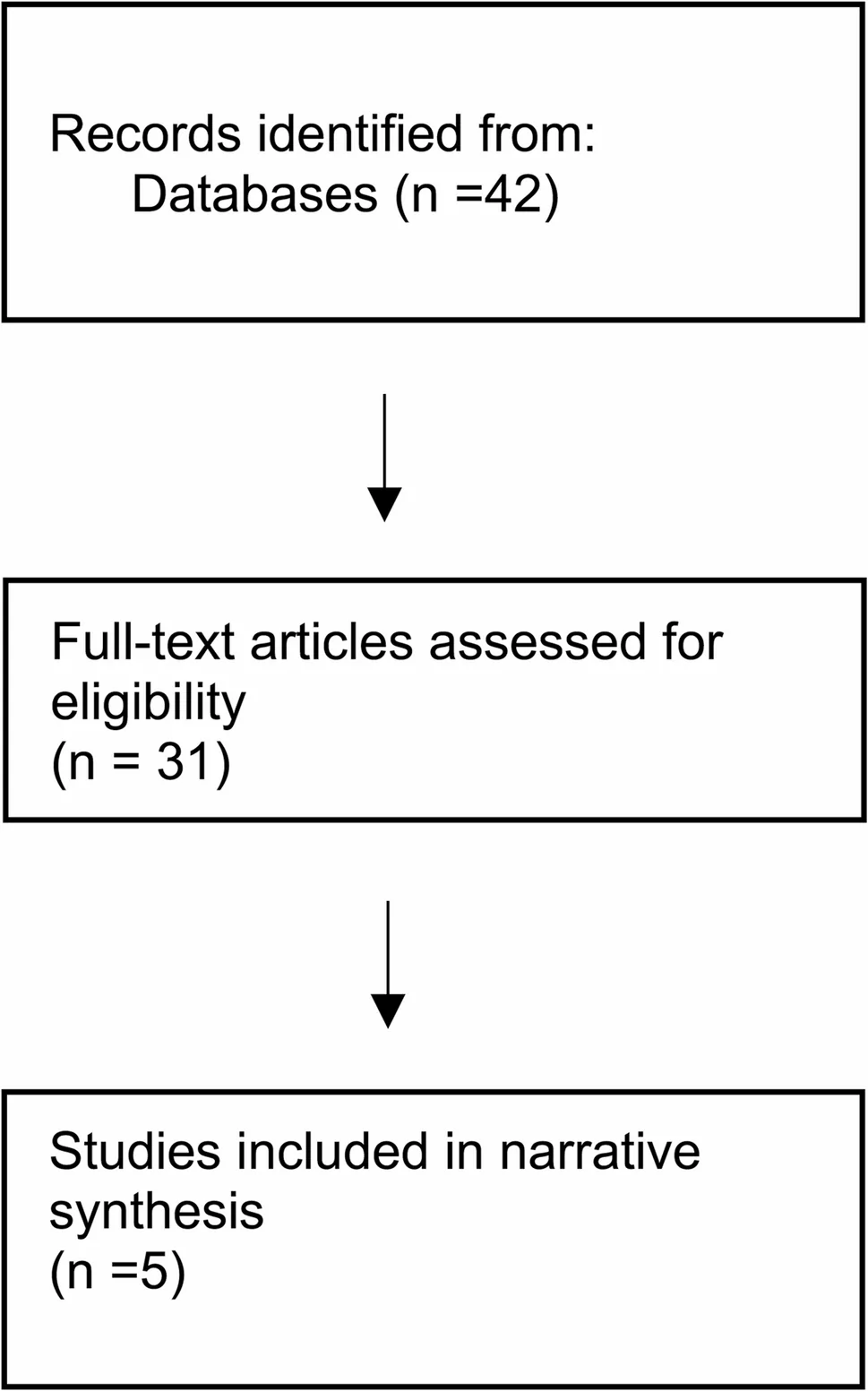
Study selection flow diagram

Methodological quality was assessed descriptively, with emphasis on model relevance, exposure conditions, and reported outcomes, based on predefined evaluation criteria including study design, reporting of cannabinoid composition, and overall methodological transparency. Two independent reviewers screened all records for eligibility to minimize selection bias. Any discrepancies between reviewers were resolved through discussion and consensus. Following full-text review and the removal of duplicates, studies with insufficient data, and non-English publications, 31 studies were identified for potential inclusion. After applying predefined exclusion criteria, five studies met the inclusion requirements and were incorporated into the final narrative synthesis (Fig. 1).

## The overview of the endocannabinoid system

The endocannabinoid system (ECS) is a multifunctional homeostatic system involved in a wide range of physiological and pathological processes (Luschnig and Schicho 2019). The ligands of the ECS are endocannabinoids (eCBs), whose actions are mimicked by exogenous cannabinoids, such as phytocannabinoids and synthetic cannabinoids (Tripathi et al. 2026). The ECS includes classical cannabinoid receptors (CB1 and CB2), as well as ECS-related receptors, G protein-coupled receptors (GPRs) 18 and 55, transient receptor ion channels (TRPV1), and nuclear peroxisome proliferator-activator receptors *(PPARs)*, all of which are responsible for mediating ECS responses (Erhabor et al. 2024). The production of eCBs occurs “on demand” from membrane phospholipid precursors. This synthesis is facilitated by canonical enzymes, namely N-acyl transferase (NAT) and N-acyl phosphatidylethanolamine phospholipase D (NAPE-PLD), which are responsible for the production of N-arachidonoylethanolamine (AEA) (Schmid et al. 2000). In contrast, the formation of 2-arachidonoylglycerol (2-AG) requires a two-step pathway: the first step involves hydrolysis by phospholipase C-β (PLC-β), followed by a second reaction catalyzed by diacylglycerol lipase (DAGL) (Bisogno et al. 2003). Once synthesized, these lipid molecules are metabolized by fatty acid amide hydrolase (FAAH) and monoacylglycerol lipase (MAGL). This enzymatic process results in the release of arachidonic acid (AA) and ethanolamine or glycerol, depending on whether AEA or 2-AG is being metabolized, respectively (Iozzo et al. 2021).

### Expression patterns of ECS components in cervical cancer models

#### Cannabinoid receptors (CNRs) and TRP channels

Cannabinoid receptors (CNRs) are expressed in female reproductive tissues and accumulating evidence suggests that the endocannabinoid system (ECS) plays a pivotal role in regulating key physiological processes (Walker et al. 2019). Studies by Popescu-Spineni et al. (2022) and Taylor et al. (2020) further indicate that the ECS may be involved in the modulation of inflammation, immune responses, and cellular proliferation, all of which are critical pathways implicated in cancer development and progression.

Analysis of the studies included in this review revealed consistent dysregulation of endocannabinoid system (ECS) components in cervical cancer cell lines and ex vivo tissues (Table 1). Contassot et al. (2004) reported of CB1, CB2 and TRPV1 in cervical cancer cell lines (HeLa, CaSki, C299) as well as in ex vivo tumor biopsy samples. Similarly, Ramer and Hinz (2008) confirmed the expression of these receptors in HeLa and C33A cervical cancer cell lines.

**Table 1:** Expression of the ECS receptors in cervical tissues and cell lines

Receptor	Normal cervical tissue	Cancer tissue/cell lines	References
CB1	Not reported	expressed	(Contassot et al. 2004; Ramer and Hinz 2008)
CB2	Not reported	expressed	(Contassot et al. 2004; Ramer and Hinz 2008)
TRPV1	Not reported	expressed	(Contassot et al. 2004; Ramer and Hinz 2008)
GPR18/55	under expressed	expressed	(Moreno et al. 2020)

A similar pattern was observed for transient receptor potential vanilloid 1 (TRPV1), which has been reported to be upregulated in cervical cancer models (Contassot et al. 2004; Ramer and Hinz 2008). TRPV1 is a non‑selective cation channel primarily localized to the plasma membrane, and its activation triggers Ca²⁺ influx, highlighting a close interplay between TRPV1 signaling and intracellular Ca²⁺ regulation, which is critical for processes such as cell migration, cytotoxicity, and apoptosis (Liu et al. 2026). Contassot et al. (2004) further demonstrated that AEA induced apoptosis in cervical cancer cell lines via aberrantly expressed vanilloid receptor-1, whereas AEA binding to the classical CB1 and CB2 cannabinoid receptors mediated a protective anti-apoptotic effect. Collectively, these findings highlight a complex, context-dependent role of cannabinoid receptors in cervical cancer models and warrants for further investigation to clarify receptor-specific signaling mechanisms.

#### Orphan cannabinoid-related receptors

Using STRING-based network analysis, Moreno et al. (2020) identified a significant expression of GPR18 and GPR55 in cervical squamous cell carcinoma compared with normal cervical tissues (Table 1). Although these findings are derived from in silico analysis rather than direct experimental models, computational approaches provide valuable system‑level insights and generate testable hypotheses that complement preclinical evidence.

Endocannabinoids also act as ligands that can bind to orphan G protein-coupled receptors (GPRs), where they may either activate or inhibit receptor-mediated signaling pathways. Among these, GPR55 (Ross 2009) and GPR119 (Rezende et al. 2023) have been identified as functionally responsive to endocannabinoids or related lipid mediators. Furthermore, increasing evidence implicates orphan GPRs in oncogenic signaling networks that regulate tumor cell proliferation, migration, angiogenesis, metabolic adaptation, and immune modulation (Chaudhary and Kim 2021; Iozzo et al. 2021; Shen et al. 2025).

GPR55 has emerged as a pro-tumorigenic receptor activated by both endogenous and exogenous cannabinoids. Its expression has been documented in multiple malignancies, including breast, glioblastoma, pancreatic, colon, and ovarian cancers. Functionally, GPR55 activation promotes tumor cell proliferation, migration, angiogenesis, and is frequently associated with poor prognosis (Ferro et al. 2018; Kargl et al. 2016; Ristić et al. 2025; Wang et al. 2023). In contrast, GPR18 has been implicated in immune cell migration, inflammation resolution, neuroprotection, and possibly antitumor effects (Honkisz-Orzechowska et al. 2024; Mahardhika et al. 2024). Although these receptors lie outside the canonical CB1/CB2 signaling framework, dysregulation of GPR55 and GPR18 substantially broadens the functional relevance of the endocannabinoid system in cancer biology, particularly with respect to tumor–immune interactions and angiogenic signaling. Their altered expression in cervical cancer suggests that malignant cells may exploit ECS‑associated pathways to support growth, survival, and immune evasion.

Importantly, such alterations also present therapeutic opportunities. Targeting orphan cannabinoid‑related receptors using phytocannabinoids or selective ECS modulators may enable restoration of dysregulated signaling networks and induction of antitumor responses. However, mechanistic validation in well‑defined in vitro and in vivo cervical cancer models is required to delineate receptor‑specific functions and to assess the translational potential of ECS‑based therapeutic strategies.

## *Cannabis sativa* phytochemistry and extraction methods

**Fig. 2:**
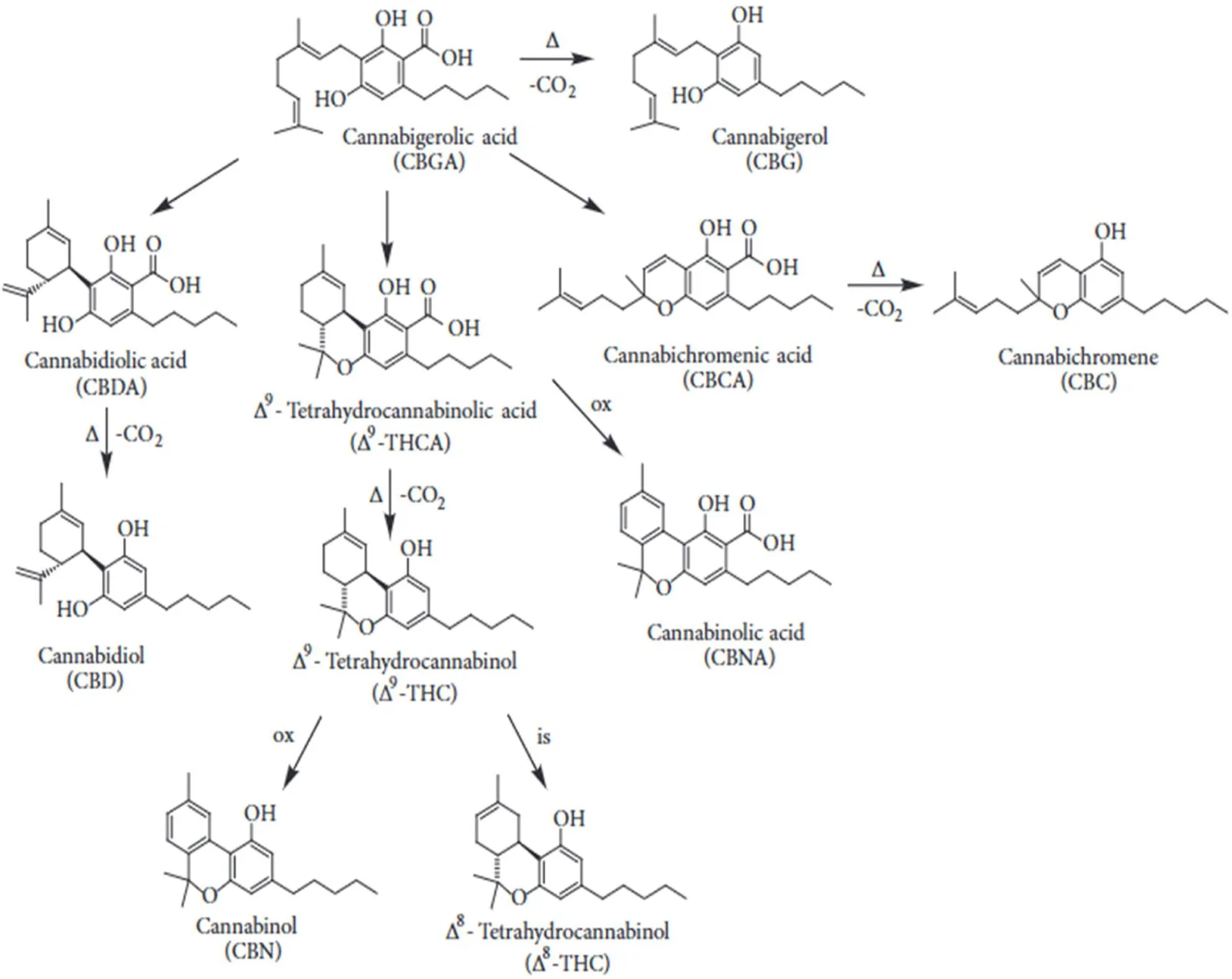
Chemical structures of the main cannabinoids present in *C. sati*va L. Abbreviations: Δ = heating; ox = oxidation; is = isomerization (Pellati et al. 2018)

*C. sativa* belongs to the Cannabaceae family and has been shown through chemical profiling to contain a wide range of phytochemicals (Monthony et al. 2021). Trichomes are specialized glandular structures present on both male and female *Cannabis sativa* plants; however, they occur at substantially higher density on the inflorescences of female plants. These structures are the primary sites for the biosynthesis and accumulation of a diverse class of terpenophenolic compounds known as phytocannabinoids (Alberti et al. 2025). These molecules exhibit tricyclic, bicyclic, and monocyclic molecular structures (Fig. 2). In most cannabis chemovars, the predominant pCBs are the acidic (carboxylic) precursors of Δ9-tetrahydrocannabinol (THC) and cannabidiol (CBD), which are decarboxylated into their active forms through drying or heating (Gülck and Møller 2020). Among the most abundant compounds are THC and its analogs (e.g., Δ8-THC and Δ9-tetrahydrocannabivarin), CBD and related compounds (e.g., cannabidivarin), cannabinol and its derivatives (e.g., cannabivarin), and cannabigerol analogs. Moreover, minor cannabinoids such as cannabinodiol, cannabichromene, cannabicyclol, cannabielsoin, and cannabitriol are also present in trace amounts (Messina et al. 2015; Pellati et al. 2018; Radwan et al. 2021).

Table 2 provides a comparative analysis of *C. sativa* extraction methods, cannabinoid and terpene composition, and strain origin. In a study conducted by Lukhele and Motadi (2016), a combination of organic solvents and ethanol was used for phytochemical extraction, with CBD identified as a major compound. A separate study, utilizing ethanol extraction followed by decarboxylation, identified both CBD and its acidic precursor, CBDA (Petrovici et al. 2021).

Supercritical carbon dioxide extraction yielded a broader cannabinoid profile, including CBD, THC, and cannabinol (CBN), demonstrating its efficiency in preserving multiple bioactive compounds (Grijó et al., 2019). Similarly, Safir et al. (2025) reported that decarboxylated *C. sativa* prior to ethanol extraction enabled the detection of CBD and THC, along with bioactive terpenes such as humulene and stigmasterol, which possess anti-inflammatory and anticancer properties. However, other studies did not report on terpene composition, thereby limiting comprehensive assessment of the full phytochemical profile and its potential contribution to therapeutic efficacy. Overall, these findings highlight the need for standardized extraction protocols and comprehensive phytochemical reporting to accurately evaluate the therapeutic potential of *C. sativa*, particularly for oncological applications.

**Table 2:** Comparative analysis of *Cannabis sativa* extraction methods, cannabinoid, terpene composition, and strain origin

Source of the plant material	Extraction method	Major cannabinoids	Major Terpenes	Reference
South Africa	Solvent extraction (butanol, n-hexane and ethanol)	CBD	Not reported	(Lukhele and Motadi 2016)
Romania	Solvent extraction (ethanol) followed by decarboxylation	CBD and CBDA	Not reported	(Petrovici et al. 2021)
-	Decarboxylation followed by Supercritical carbon dioxide extraction (scCO_2_)	CBD, THC and CBN	Not reported	(Grijo et al. 2019)
Pakistan	Solvent extraction (ethanol)	CBD and THC	Humulene and Stigmasterol	(Safir et al. 2025)

### The need for standardization in cannabis‑based research

Cannabinoid composition in *Cannabis sativa* is influenced by a combination of genetic, environmental, and processing factors. Genotype (chemovar) is the primary determinant of the qualitative cannabinoid profile (e.g., THC‑ or CBD‑dominant), although quantitative levels can vary significantly within the same genotype depending on cultivation conditions (Danziger and Bernstein 2021; Naim-Feil et al. 2023; Trancoso et al. 2022). Environmental and agronomic variables such as light, temperature, soil composition, irrigation, plant developmental stage, and harvest timing contribute to substantial batch‑to‑batch variability, even within the same chemotype. This variability can affect reproducibility in research and highlights the need for rigorous batch characterization using analytical methods like HPLC or GC-MS (Cicaloni et al. 2022; Xiong et al. 2013).

Chemotype classifications (THC‑dominant, CBD‑dominant, or balanced) provide a useful framework but do not fully capture the complexity of cannabis phytochemistry, as minor cannabinoids, terpenes, and flavonoids may also significantly influence biological activity through the so‑called entourage effect (Mayong et al. 2026; Russo 2019). Consequently, whole‑plant extracts may exhibit different pharmacological effects compared to isolated cannabinoids, underscoring the importance of comprehensive phytochemical profiling (André et al. 2024).

Standardization is therefore essential to ensure reproducibility and comparability across studies, requiring controlled cultivation practices, consistent harvesting protocols, reproducible extraction methods, and adherence to GACP and GMP guidelines, along with the use of certified reference materials (Costa et al. 2025; Ilikj et al. 2021; Malabadi et al. 2025). Additionally, extraction and processing conditions such as solvent choice (e.g., ethanol, hydrocarbons, or supercritical CO₂) and steps like decarboxylation, winterization, and distillation can significantly alter the chemical profile and stability of cannabis-derived products, further emphasizing the need for careful optimization and transparent methodological reporting (Costa et al. 2025; Rochfort et al., 2020).

## Anticancer potential, mechanisms and signaling pathways of Cannabis sativa in cervical cancer

### Overview of preclinical evidence: in vitro

All included studies were conducted exclusively in vitro using cervical cancer cell lines. While these models provide mechanistic insights, they do not replicate in vivo conditions such as tumor microenvironment, immune interactions, or pharmacokinetics. Notably, two of the five included studies did not investigate molecular mechanisms, which limits the depth of mechanistic interpretation. While in vitro studies are essential for preliminary efficacy assessment and mechanistic investigation, their findings should be validated in appropriate in vivo models before meaningful translational implications can be established (Steger-Hartmann and Raschke 2020).

Table 3 summarizes preclinical studies investigating the effects of *C. sativa* and its phytocannabinoids in cervical cancer models. Among the five studies included, four examined full-spectrum *C. sativa* oil extracts, and one focused specifically on purified cannabidiol (CBD). Full-spectrum *C. sativa* oil extracts comprising a wide range of phytocannabinoids, terpenes, and flavonoids have demonstrated notable anticancer activity in preclinical cancer models (Bukowska 2024; Tomko et al. 2020). Unlike purified compounds, full-spectrum preparations retain the plant’s natural chemical diversity, potentially enabling synergistic effects that enhance therapeutic efficacy (André et al. 2024).

Furthermore, methodological limitation across the included in vitro studies is the widespread use of serum‑supplemented culture media. Cannabinoids are highly lipophilic and extensively bind serum proteins such as albumin, serum inclusion substantially reduces the unbound, bioactive fraction available to cells, thereby altering effective exposure and observed cytotoxicity. Variability in serum type and concentration across studies is therefore likely to contribute to inconsistencies in reported IC₅₀ values, limiting inter‑study comparability and complicating the extrapolation of in vitro potency data to in vivo systems (Carkaci-Salli et al. 2024).

### Anticancer effects of*Cannabis sativa*extracts in cervical cancer cell lines

A study conducted by Grijo et al. (2019) revealed that supercritical carbon dioxide extraction of *C. sativa* oil effectively preserved both cannabinoid and terpene contents. Notably, one of the extracts demonstrated strong cytotoxicity against the HeLa, SiHa, and C33A cell lines, with IC_50_ values ≤ 3.1 µg/mL and high selectivity indices ranging from 25.9 to 48.9. As proposed by Indrayanto et al. (2021), samples with a selectivity index of ≥ 10 are considered sufficiently selective and may be regarded as promising candidates for further preclinical evaluation.

Safir et al. (2025) reported that *C. sativa* extracts (IC_50_= 49 µg/mL) induced apoptosis in HeLa cell lines. Petrovici et al. (2021) further demonstrated that CBD-enriched hemp oil induced oxidative stress by increasing intracellular hydrogen peroxide (H₂O₂) levels and altering the antioxidant defense system, specifically downregulating catalase (CAT) and upregulating glutathione peroxidase 1 (GPX1). This extract reduced HeLa cell viability with an IC_50_ of 16.89 µg/mL. Interestingly, apoptosis appeared to be p53-independent, as TP53 expression was suppressed. Furthermore, Petrovici et al. (2021) reported a BAX/BCL2 ratio of < 1, which typically indicates resistance to classical mitochondrial (intrinsic) apoptosis, yet this finding was observed alongside a significant reduction in cell viability. This apparent contradiction suggests that cell death occurred despite incomplete or ineffective activation of canonical apoptotic pathways, highlighting a disconnect between molecular apoptosis markers and phenotypic cytotoxic outcomes. One plausible explanation is that CBD-induced cytotoxicity may involve non-apoptotic or mixed cell death mechanisms, such as oxidative stress-mediated damage, mitochondrial dysfunction, or caspase-independent cell death pathways.

Accumulating preclinical evidence further highlights the anticancer potential of *C. sativa*-derived phytochemicals, particularly cannabidiol (CBD) and Δ⁹-tetrahydrocannabinol (Δ⁹-THC). These bioactive compounds exert multifaceted antitumor effects, including the induction of apoptosis, inhibition of cellular invasion, modulation of oxidative stress, and the disruption of tumor-supportive signaling pathways (Leelawat et al. 2022; Pongking et al. 2024; Todorova et al. 2021). Earlier studies by Contassot et al. (2004) demonstrated that the endocannabinoid anandamide (AEA) induces apoptosis in cervical cancer cells (HeLa, CaSki, CC299) via activation of the transient receptor potential vanilloid 1 (TRPV1) channel, leading to caspase-7 activation and DNA fragmentation. Notably, CB1 and CB2 receptor antagonism enhanced this apoptotic response, suggesting that these receptors may play a protective role in cervical cancer cells. Activation of CB1 and CB2 receptors has been demonstrated across multiple cancer models to engage pro-survival signaling pathways, particularly the phosphoinositide 3-kinase (PI3K)/Akt and mitogen-activated protein kinase/extracellular signal-regulated kinase (MAPK/ERK) cascades, which are central regulators of cell proliferation, survival, and resistance to apoptosis. Upon receptor activation, downstream signaling can lead to phosphorylation of Akt, promoting the inactivation of pro-apoptotic proteins such as Bad and activation of anti-apoptotic regulators, thereby enhancing cell survival (Velasco et al., 2012; Afrin et al., 2020; Dando et al., 2013). Similarly, CB1/CB2-mediated activation of the ERK pathway has been shown to drive transcriptional programs associated with cell proliferation and survival, contributing to tumor progression under certain conditions (Blázquez et al., 2006; Sarfaraz et al., 2008). Notably, these effects are highly context-dependent, as cannabinoids have also been reported to inhibit PI3K/Akt and ERK signaling in other settings, leading to apoptosis rather than survival (Velasco et al., 2016; Moreno et al. 2019).

This bidirectional behavior is particularly relevant in cervical cancer, where Contassot et al. (2004) demonstrated that CB1/CB2 receptor antagonism enhanced apoptosis, implying that receptor activation may act to counterbalance pro-apoptotic signaling, particularly that mediated via TRPV1. Such findings suggest that ECS signaling is not inherently antitumorigenic but can instead promote tumor cell survival depending on receptor context, ligand, and intracellular signaling environment. This complexity reinforces the importance of receptor-specific and pathway-specific investigation, rather than generalized assumptions regarding cannabinoid effects in cancer.

Further evidence from Lukhele et al. (2016) revealed that CBD induced apoptosis in HeLa, SiHa, and ME-180 cell lines via activation of p53, upregulation of proapoptotic Bax, downregulation of pro-apoptotic Bcl-2, and cleavage of caspase-3 and − 9, with a concomitant reduction in intracellular ATP. Importantly, CBD exhibited greater potency (IC_50_ = 1.5–3.2 µg/mL) compared to *C. sativa* extracts, indicating the therapeutic potential of isolated phytocannabinoids (Lukhele and Motadi 2016).

While these studies provide valuable mechanistic insights into cannabinoid‑mediated molecular pathways under controlled laboratory conditions, the absence of in vivo validation limits conclusions regarding tumor progression, systemic effects, and clinical efficacy.

**Table 3:** Summary of preclinical studies on the effects of *Cannabis sativa* and its phytocannabinoids on cervical cancer models

Study	Model	Mechanism	Outcome	Authors
Cannabidiol inhibits cell growth in cervical cancer cells.	HeLa, SiHa and ME-180 cell lines	Apoptosis induction via p53 activation, caspase-3/9 upregulation, Bax upregulation, Bcl-2 downregulation, and ATP depletion.	CBD* (IC_50_ =1.5–3.2 µg/mL) Induced significant apoptosis and cell death in all cell lines, outperforming crude *C. sativa* extracts (IC_50_= 50–100 µg/mL).	(Lukhele et al.,^2016^)
Cannabidiol inhibits cancer cell invasion via upregulation of tissue inhibitor of matrix metalloproteinases-1	HeLa and C33A cell lines	Anti-invasive effects via upregulation of TIMP-1, mediated by p38 and p42/44 MAPKs. Effects reversed by CB1/CB2/TRPV1 antagonists or TIMP-1 knockdown.	CBD (10µM) significantly inhibited tumor invasion in vitro	(Ramer et al. 2010)
New Insights on Hemp Oil Enriched in Cannabidiol: Decarboxylation, Antioxidant Properties and In Vitro Anticancer Effect	HeLa cell lines	Apoptosis induction via ROS production. Downregulation of catalase (CAT) and upregulation of glutathione peroxidase 1 (GPX1). p53-independent pathway (TP53 gene downregulated).	Reduced cell viability (IC_50_ = 16.89 µg CBD/mL). Increased H_2_O_2_ production (oxidative stress). Morphological changes (cytoplasmic condensation, cell detachment). Resistance to apoptosis (BAX/BCL2 ratio < 1) but significant cell death at higher doses.	(Petrovici et al. 2021)
Analysis of the antitumor activity of bioactive compounds of Cannabis flowers extracted by green solvents	HeLa, SiHa and C33 cell lines	Mechanism not investigated	Extract #5, obtained via supercritical CO₂ extraction (40 MPa, 50 °C, 30 min decarboxylation), exhibited potent antitumor activity against cervical cancer cells (IC_50_ ≤ 3.1 µg/mL) with high selectivity (indices 25.9–48.9) due to its elevated Δ⁹-THC content (68.3%).	(Grijo et al. 2019)
Extraction, GC-MS analysis, cytotoxic, anti-inflammatory and anticancer potential of *Cannabis* *sativa* female flower; in vitro, in vivo and in silico	HeLa	Apoptosis	*C. sativa* extracts (IC_50_= 49 µg/mL) induced increased apoptosis in cervical cancer cells.	(Safir et al. 2025)

“Not investigated” indicates that the study did not assess or report molecular mechanisms. The absence of mechanistic data represents a significant limitation, as mechanistic conclusions are based on a subset of included studies

CBD* purchased

### Mechanisms and signaling pathways implicated in cervical cancer

Cancer is a heterogeneous disease, and current evidence must be interpreted with consideration of the variability in ECS components across different tumor types. Numerous preclinical studies and histological analyses of patient tumors indicate that the upregulation of CB1 and CB2 receptors, along with increased levels of endogenous ligand levels and overactivation of the ECS is associated with more aggressive tumor phenotypes (Dariš et al. 2019). The functional roles of endogenous cannabinoids and their receptors appear to be context-dependent, varying according to the specific cancer type, which may account for the conflicting findings reported in the literature. Furthermore, some cannabinoids have demonstrated antitumor effects that are independent of known CB receptors, suggesting the potential involvement of yet unidentified CB receptors in cancer pathophysiology (Moreno et al. 2019).

**Fig. 3:**
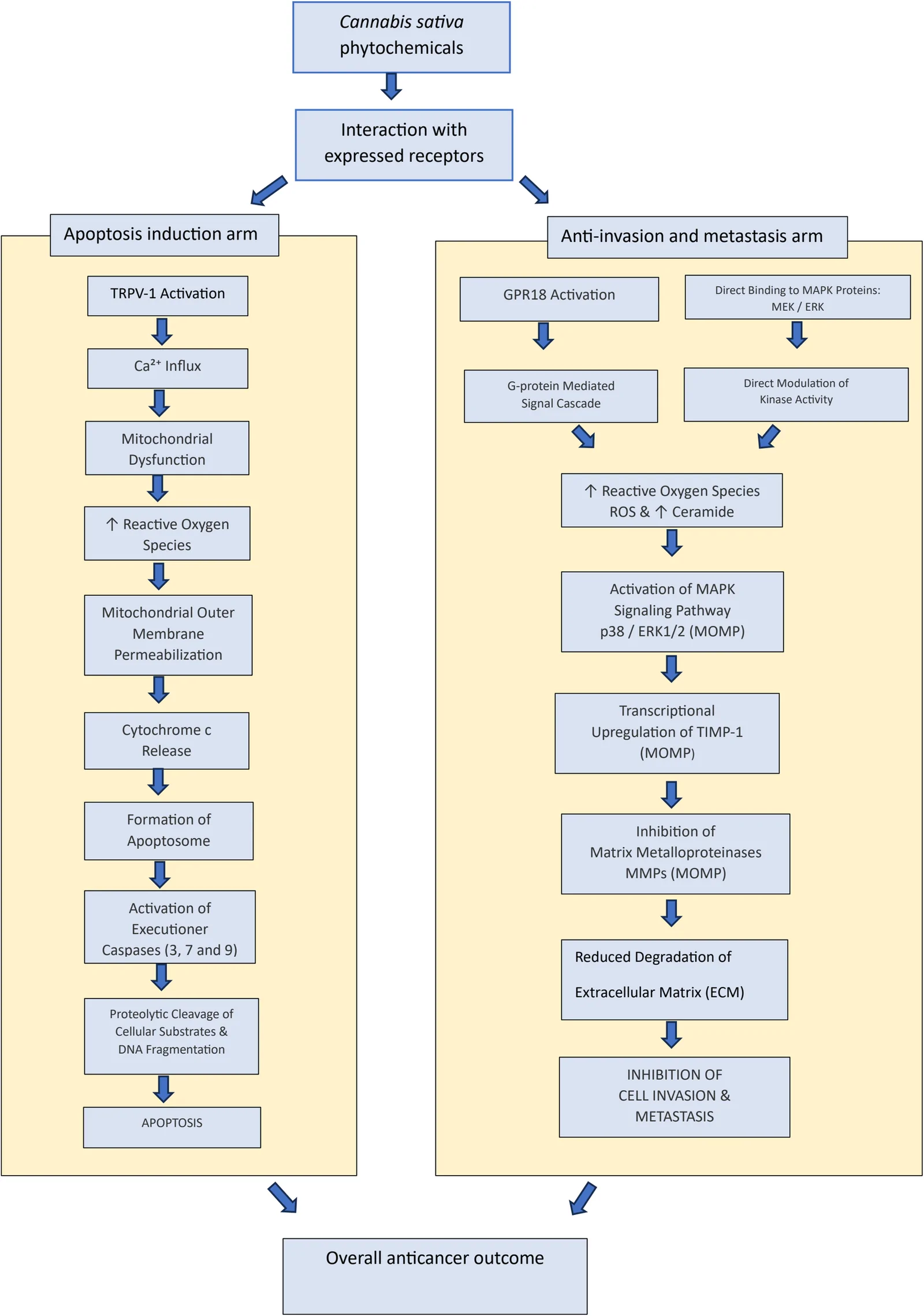
Overview of the proposed molecular mechanism by which *Cannabis sativa* phytochemicals exert anticancer effects in cervical cancer models, delineating two primary signaling arms that converge to inhibit tumor progression. In the apoptosis induction arm, activation of transient receptor potential vanilloid 1 (TRPV1) by endocannabinoids like anandamide (AEA) or phytocannabinoids such as cannabidiol (CBD) triggers Ca²⁺ influx, leading to mitochondrial dysfunction. This results in the production of reactive oxygen species (ROS) and the accumulation of proapoptotic lipid ceramide. These stressors induce mitochondrial outer membrane permeabilization (MOMP), prompting the release of cytochrome c into the cytosol. Cytochrome c facilitates the formation of the apoptosome complex, which activates the initiator caspase-9. Caspase-9, in turn, cleaves and activates the executioners caspases-3 and − 7, culminating in proteolytic degradation of cellular components and DNA fragmentation, thereby executing programmed cell death. Simultaneously, the anti-invasion and metastasis arm is initiated by the activation of the orphan G-protein coupled receptor GPR18 by CBD and the direct binding of phytocannabinoids such as CBD and Δ9-tetrahydrocannabinol (Δ9-THC) to mitogen-activated protein kinase (MAPK) pathway components, as supported by in silico docking data. These interactions stimulate the MAPK signaling cascade, specifically activating p38 and extracellular signal-regulated kinases 1/2 (ERK1/2). Once activated, these kinases translocate to the nucleus, where they phosphorylate and activate downstream transcription factors, leading to upregulation of tissue inhibitor of metalloproteinases‑1 (TIMP‑1) expression. The secreted TIMP-1 protein inhibits the enzymatic activity of matrix metalloproteinases (MMPs), which are required for the degradation of the extracellular matrix (ECM). This inhibition prevents ECM breakdown, thereby blocking cancer cell invasion and metastatic spread. The coordinated action of these two arms—inducing apoptosis and suppressing invasion—results in the overall inhibition of cervical tumor growth and metastasis. Abbreviations: TRPV1, transient receptor potential vanilloid 1; GPR18, G protein-coupled receptor 18; MAPK, mitogen-activated protein kinase; MEK, mitogen-activated protein kinase; ERK, extracellular signal-regulated kinase; ROS, reactive oxygen species; MOMP, mitochondrial outer membrane permeabilization; TIMP-1, tissue inhibitor of metalloproteinases-1; MMPs, matrix metalloproteinases; ECM, extracellular matrix; CBD, cannabidiol; Δ9-THC, Δ9-tetrahydrocannabinol; AEA, anandamide

As illustrated in Fig. 3, the proposed mechanisms through which *Cannabis sativa* phytochemicals influence cervical cancer cell behavior involve multiple intersecting signaling pathways, primarily derived from in vitro data. While cannabinoids have been widely studied across different cancer models, it is important to note that mechanistic evidence in cervical cancer remains limited and largely restricted to cell culture systems. Cannabinoids such as Δ9-tetrahydrocannabinol (Δ9-THC) and cannabidiol (CBD) have been reported to modulate key features associated with cancer biology, including apoptosis, oxidative stress, and cell invasion (Massi et al., 2013; Younes et al., 2024). . Δ9-tetrahydrocannabinol (Δ9-THC) predominantly induces apoptosis and cytotoxicity through cannabinoid receptor-dependent pathways, whereas CBD exerts its effects by signaling mechanisms involving orphan G protein-coupled receptors (GPCRs) and non-GPCR-mediated pathways (Velasco et al., 2012; 2016; Afrin et al., 2020).

Δ⁹-THC acts as a partial agonist at cannabinoid receptors CB1 and CB2, and receptor activation is known to initiate signaling cascades that regulate cancer cell fate. These include induction of apoptosis through ceramide accumulation, endoplasmic reticulum stress, and mitochondrial dysfunction, as well as inhibition of pro-survival pathways such as PI3K/Akt and MAPK/ERK (Greenhough et al. 2007; Pagano et al. 2021). The high Δ⁹-THC content of the extract (Grijo et al. 2019) likely amplifies these receptor-mediated effects, thereby contributing to the observed cytotoxic potency and selectivity in vitro.

Cervical cancer development and progression are governed by intricate molecular signaling networks that regulate cellular proliferation, survival, invasion, angiogenesis, and resistance to apoptosis. Among these, the phosphoinositide 3-kinase (PI3K)/Akt pathway, mitogen-activated protein kinase (MAPK) cascade, and nuclear factor kappa-light-chain-enhancer of activated B cells (NF-κB) signaling axis play dominant roles in oncogenic transformation and immune evasion but also position ECS components as promising molecular targets in cervical cancer (Braicu et al. 2019; Peng et al. 2022; Zhang et al. 2015). Ramer et al. (2010) showed that CBD significantly upregulates TIMP-1 expression in HeLa and C33A cervical cancer cells. This upregulation was mechanistically dependent on MAPK signaling, as pharmacological inhibition of p38 and extracellular signal-regulated kinases (ERK1/2) abrogated TIMP-1 expression. The anti-invasive effects of CBD were also reversed upon TIMP-1 knockdown via siRNA, establishing a causal link between CBD-induced MAPK activation, TIMP-1 expression, and the suppression of cancer cell invasiveness. Consistent with these findings Ngnameko et al. (2026), reported that *C. sativa* inhibited migration and invasion by downregulating MMP-1, MMP-9 in MDA-MB-231 (IC_50_ 75.46 ± 0.132 µg/mL) and MCF-7 (IC_50_ 78.68 ± 0.50 µg/mL) cancer cell lines. While Solinas et al. (2013), demonstrated in human glioma cell lines U87-MG (IC_50_ 13.41µM) and T98G (IC_50_ 13.41µM) that CBD downregulated matrix metalloproteinase MMP-9, tissue inhibitors of metalloproteinase TIMP-1 and TIMP-4.

The interaction of cannabinoids with ECS receptors further underscores their impact on cervical cancer signaling. Contassot et al. (2004) showed that the endocannabinoid anandamide (AEA) induces apoptosis in HeLa, CaSki, and CC299 cervical cancer cells primarily through activation of transient receptor potential vanilloid 1 (TRPV1), a non‑selective cation channel. TRPV1 activation resulted in Ca²⁺ influx, caspase‑7 activation, and DNA fragmentation, while pharmacological blockade of TRPV1 abrogated AEA‑induced cell death, confirming its essential role in the pro‑apoptotic response.

Reactive oxygen species (ROS) generation represents an additional and well-characterized mechanism underlying cannabinoid-mediated anticancer activity. ROS are unstable oxygen-derived molecules that induce oxidative stress, leading to mitochondrial dysfunction, DNA damage, and apoptosis. Several studies, including those by Razlog et al. (2013), have demonstrated that CBD treatment elevates intracellular ROS levels in cervical cancer cells. This increase in oxidative stress, in turn, activates intrinsic apoptotic pathways via mitochondrial membrane depolarization, cytochrome c release, and caspase-3/9 cleavage (Nakamura and Takada 2021). Inhibitors of ROS have been shown to reverse CBD-induced apoptosis, confirming that ROS generation is a central mechanism of action (Shrivastava et al. 2011). Furthermore, CBD has been reported to induce the accumulation of ceramide, a lipid-based signaling molecule that promotes mitochondrial stress and facilitates caspase activation (Massi et al. 2008).

Taken together, cumulative evidence illustrates that *C. sativa* phytochemicals exert anti-cervical cancer effects by disrupting multiple oncogenic signaling pathways. CBD modulates MAPK signaling to induce TIMP-1 expression and inhibit invasion, increases ROS to trigger mitochondrial dysfunction, and interacts with CB1, CB2, and TRPV1 receptors to orchestrate anti-apoptotic responses (Heider et al. 2022; Seltzer et al. 2020). This is strongly supported by in silico evidence demonstrating that cannabinoids, including CBD and THC, have high binding affinities for key proteins in the MAPK-ERK pathway, suggesting a direct mechanism for their modulatory effects (Iqbal and Matsabisa 2024). Furthermore, computational pharmacokinetic analyses confirmed that these cannabinoids possess favorable drug-likeness properties, including high gastrointestinal absorption and the ability to act as substrates for metabolic enzymes such as CYP450, which supports their potential as viable therapeutic agents (Iqbal and Matsabisa 2024). The complexity of these interactions presents both an opportunity and a challenge for cannabinoid-based therapy. While the ability to target multiple signaling pathways is advantageous in combating tumor heterogeneity and resistance, it also necessitates precision in drug design to ensure the selective activation or inhibition of ECS components. Future studies must focus on mapping the signaling landscape of ECS receptors in cervical cancer subtypes and elucidating the temporal dynamics of receptor-ligand interactions, building upon the structural insights provided by molecular docking studies. In parallel, in vivo validation and early-phase clinical trials are essential to translate these molecular and computational insights into tangible treatment strategies. 

### *C. sativa*phytochemicals and HPV associated cancers

To date, no studies have directly evaluated the effects of C. sativa phytochemicals in HPV-driven cervical cancer models, representing a critical limitation of the available evidence. Consequently, any interpretation of HPV-related mechanisms must be approached with caution. HPV-associated carcinogenesis is driven by the viral oncoproteins E6 and E7, which promote degradation of tumor suppressors p53 and retinoblastoma (pRb), resulting in unchecked proliferation, genomic instability, and resistance to apoptosis (Braicu et al. 2019; Dariš et al. 2019). Sen et al. (2025) demonstrated both in vitro and in vivo that CBD suppressed proliferation and migration of HPV‑positive HNSCC cells by inducing apoptosis via activation of the MAPK pathway, including ERK1/2, JNK/SAPK, and MK2. In contrast, Liu et al. (2020) demonstrated that cannabinoids promote the progression of HPV-positive HNSCC via p38 MAPK activation using in vitro and animal models. These findings highlight a clear mechanistic contradiction, as cannabinoid signaling is associated with both tumor-suppressive and tumor-promoting effects within similar disease contexts. This divergence suggests that cannabinoid-mediated modulation of signaling pathways, including MAPK, is highly context-dependent, influenced by factors such as receptor expression, ligand specificity, and intracellular signaling networks. While cannabinoids have been reported to induce apoptosis through both p53-dependent and p53-independent mechanisms, the opposing outcomes observed across studies indicate that their net biological effect cannot be generalized as uniformly antitumorigenic.

## Limitations

While this review highlights promising preclinical findings, several important limitations should be considered. Most notably, the available evidence is derived solely from a small number of in vitro studies, as no in vivo investigations evaluating *Cannabis sativa* or its phytocannabinoids in cervical cancer models were identified. Although in vitro models are valuable for exploring cellular responses and underlying molecular mechanisms, they cannot fully reproduce the complexity of living systems, including pharmacokinetic processes, the tumor microenvironment (TME), immune interactions, and systemic biological effects (Iozzo et al. 2021). Additionally, significant methodological heterogeneity exists across the included studies. Differences in *C. sativa* chemotypes, extraction methods, cannabinoid composition and concentrations, as well as the cervical cancer cell lines employed (e.g., HeLa, SiHa, and C33A), limit direct comparisons between studies and complicate the interpretation and generalizability of findings.

### Conclusion and future directions

This review synthesizes the currently available evidence on Cannabis sativa phytochemicals and the endocannabinoid system (ECS) in cervical cancer, highlighting a limited but mechanistically informative body of in vitro research. Collectively, the findings indicate that C. sativa-derived compounds, particularly cannabidiol (CBD) and Δ⁹‑tetrahydrocannabinol (Δ⁹‑THC), can modulate key cellular processes, including apoptosis, oxidative stress, and invasion. However, these effects are highly context-dependent and involve both cytotoxic and cytoprotective signaling pathways. Importantly, ECS signaling does not appear to be uniformly antitumorigenic. Evidence demonstrates that activation of CB1 and CB2 receptors may exert pro-survival, anti-apoptotic effects in certain cervical cancer models, while non-classical pathways, such as TRPV1 activation and ROS generation, are more consistently associated with pro-apoptotic outcomes. Furthermore, the proposed roles of orphan receptors such as GPR18 and GPR55 remain speculative, as current evidence is largely derived from in silico analyses and lacks direct experimental validation in cervical cancer systems.

The interpretation of these findings is further constrained by the small number of available studies, all of which are limited to in vitro models. Additionally, methodological heterogeneity across studies complicates direct comparison and interpretation. Taken together, the current evidence base remains preliminary and insufficient to support conclusions regarding therapeutic efficacy or clinical application. Future research should focus on well-designed in vivo studies, mechanistic investigations in HPV-associated cervical cancer models, and pharmacokinetic characterization of cannabinoids. Standardization of cannabis-derived products and rigorous evaluation of receptor-specific effects will be essential to clarify the biological role of ECS modulation and its potential relevance in cervical cancer.

## Data Availability

No datasets were generated or analysed during the current study.
